# An Antioxidative Exopolysaccharide–Protein Complex of *Cordyceps* Cs-HK1 Fungus and Its Epithelial Barrier-Protective Effects in Caco-2 Cell Culture

**DOI:** 10.3390/antiox14121501

**Published:** 2025-12-14

**Authors:** Yan Yu Zhu, Margaret M. H. Wu, Zi Chen Zhao, Fang Ting Gu, Lin Xi Huang, Kevin W. H. Kwok, Jian Yong Wu

**Affiliations:** Research Institute for Future Food, Department of Food Science and Nutrition, The Hong Kong Polytechnic University, Hung Hom, Kowloon 999077, Hong Kong; yanyu.zhu@connect.polyu.hk (Y.Y.Z.);

**Keywords:** *Cordyceps sinensis* fungus, exopolysaccharide-protein complex, antioxidant activity, intestinal barrier, Caco-2 cells

## Abstract

The exopolysaccharides (EPS) from the mycelial fermentation of *Cordyceps sinensis* Cs-HK1, especially the low-molecular weight, protein-rich exopolysaccharide fractions (EPS-LM), have previously exhibited significant antioxidant activity. This study further investigated the antioxidant and protective effects of EPS-LM on intestinal epithelial barrier integrity in Caco-2 monolayers challenged with hydrogen peroxide (H_2_O_2_, 550 μM). EPS-LM contained two major molecular-weight fractions, 25 kDa and 1.7 kDa, with 19.3% total carbohydrate and 28.7% protein content (*w*/*w*). Treatment of the cells with EPS-LM (50–200 μg/mL) showed concentration-dependent protective effects against ROS-induced losses of cell viability and epithelial barrier integrity. EPS-LM treatment enhanced the activities of major antioxidant enzymes (SOD, GSH-Px, and CAT) and modulated NRF2 and its downstream target NQO1, consistent with alleviated oxidative stress. It also improved several indicators of intestinal barrier function, including increased transepithelial electrical resistance and upregulation of tight junction proteins (Occludin, ZO-1, and Claudin-1). These results provide new experimental evidence and theoretical basis for the nutraceutical potential of EPS-LM to mitigate oxidative stress and preserve intestinal epithelial barrier integrity.

## 1. Introduction

The intestinal epithelial barrier represents the first line of host defense against luminal threats, preventing the translocation of pathogens and toxins into the internal circulation while selectively allowing nutrient and signal exchange between the host and environment [1]. Tight junctions (TJs) are central to this barrier system, regulating paracellular permeability and protecting underlying tissues from luminal contents [2]. Disruption of TJ integrity compromises epithelial protection, enabling the passage of luminal antigens and microbes. This breach provokes immune activation and chronic inflammation, which exacerbate gastrointestinal disorders including inflammatory bowel disease (IBD), irritable bowel syndrome (IBS), and “leaky gut syndrome,” and can also contribute to systemic complications such as metabolic, autoimmune, and neurological diseases [3,4].

Oxidative stress, largely driven by the overproduction of reactive oxygen species (ROS), plays a pivotal role in TJ disruption. Under physiological conditions, endogenous antioxidant systems maintain redox balance; however, during infection or inflammation, uncontrolled ROS can damage DNA, proteins, and lipids [5,6]. Excessive ROS specifically compromises TJ components such as ZO-1, Claudins, and Occludin, leading to their redistribution or degradation, elevated paracellular permeability, and increased translocation of luminal substances. This barrier breakdown amplifies inflammation and oxidative stress, generating a self-perpetuating cycle that further deteriorates epithelial integrity [7,8].

Among TJ proteins, Claudin-1, Occludin, and ZO-1 play essential structural and regulatory roles. Claudin-1 forms selective paracellular barriers between epithelial cells, controlling ion and solute permeability, while Occludin contributes to junctional sealing and stabilizes interprotein connections with the cytoskeleton [9,10]. ZO-1, a multidomain cytoplasmic scaffolding protein, anchors transmembrane proteins such as Claudin-1 and Occludin to the actin cytoskeleton and mediates dynamic regulation of junctional complexes in response to signaling cues [11,12]. Together, these TJ proteins coordinate to sustain epithelial cohesion and preserve barrier functionality.

To counteract oxidative injury, cells activate the Kelch-like ECH-associated protein 1-nuclear factor erythroid 2-related factor 2 (KEAP1-NRF2) axis, a master regulatory pathway governing antioxidant and cytoprotective responses. Upon oxidant challenge (e.g., H_2_O_2_), critical cysteine residues on KEAP1 become modified, which disrupts the KEAP1-CUL3 complex and allows NRF2 to escape ubiquitin-mediated degradation. Accumulated NRF2 translocates into the nucleus and binds to antioxidant response elements (AREs), driving the transcription of detoxifying and phase II enzymes such as NAD(P)H: quinone oxidoreductase 1 (NQO1), heme oxygenase-1 (HO-1), and glutamate-cysteine ligase catalytic subunit (GCLC). Through these effectors, the NRF2 pathway restores cellular redox balance, enhances resilience against oxidative stress, and contributes to the maintenance of epithelial integrity [13,14].

Natural polysaccharides from food and medicinal sources have been widely reported to alleviate oxidative stress and preserve epithelial structure and function, thereby maintaining intestinal homeostasis [15]. By reducing oxidative load, these polysaccharides can modulate NRF2-regulated genes and promote intracellular conditions favorable for tight junction maintenance and barrier integrity [16,17]. *Cordyceps* (*Ophiocordyceps*) *sinensis* (*C. sinensis*), a highly valued medicinal fungus in traditional Chinese medicine, exhibits multiple bioactivities including antioxidant, anti-inflammatory, and immunomodulatory effects [18]. Because natural *C. sinensis* is rare and costly, mycelial fermentation provides a sustainable and economical alternative. *C. sinensis* Cs-HK1, an anamorphic strain identified by Wu’s group, can produce mycelial biomass and exopolysaccharides (EPS) with notable biological functions [19]. The EPS released to the liquid medium during Cs-HK1 mycelial fermentation consist mainly of polysaccharide-protein complexes spanning a broad molecular weight range from several kDa to over 10,000 kDa [20]. The low-molecular weight, protein-rich exopolysaccharide fractions from Cs-HK1 (EPS-LM) have been shown enhanced antioxidant and anti-inflammatory properties [21], distinct from many of the plant- and fungus-derived polysaccharides that are predominantly high-molecular weight and carbohydrate-rich. This hybrid composition may confer dual functionality, with the polysaccharide moiety contributing to antioxidant and redox-regulating effects that have been widely described for natural polysaccharides [15,16] and the protein and amino acid components providing additional bioactivities [22]. Together, these features make EPS-LM a particularly promising candidate for targeting intestinal barrier dysfunction under oxidative stress. However, despite these attractive properties, mechanistic evidence linking EPS-LM mediated antioxidant activity to the preservation of intestinal epithelial barrier integrity under oxidative stress remains limited.

Based on the above background and our previous findings, we hypothesized that EPS-LM would protect intestinal epithelial barrier integrity under oxidative stress by attenuating ROS accumulation, enhancing cellular antioxidant defenses, and preserving TJ structure. To attest this hypothesis, this study was carried out to characterize the chemical composition of a protein-rich EPS-LM fraction, including total carbohydrate and protein contents, as well as monosaccharide composition and amino acid profiles, and evaluate its antioxidant activities using chemical assays and cell culture models to measure intracellular ROS and antioxidant enzyme activities. Furthermore, its protective effect was assessed on the transepithelial electrical resistance (TEER) and TJ protein expression in H_2_O_2_-challenged Caco-2 monolayers to elucidate potential redox-related mechanisms underlying barrier restoration.

## 2. Materials and Methods

### 2.1. Cs-HK1 Fermentation and EPS-LM Isolation

As reported previously [23], the mycelial fermentation of Cs-HK1 was performed in shake-flasks using a liquid medium containing 40 g/L glucose, 5 g/L peptone, 1 g/L KH_2_PO_4_, 0.5 g/L MgSO_4_·7H_2_O, and 10 g/L yeast extract at 20 °C for 7 days. At the end, the liquid fermentation broth was centrifuged at 12,000 rpm for 15 min, and the supernatant was collected for extraction of EPS. EPS was extracted from the liquid supernatant by a two-step ethanol precipitation as reported previously [21], using 40% (*v*/*v*) ethanol in the first step to precipitate the high-MW EPS and 80% ethanol in the second step to precipitate the low-MW EPS (EPS-LM).

### 2.2. Characterization and Analysis of EPS-LM

#### 2.2.1. Chemical Composition Analysis

The total carbohydrate content was measured using the anthrone test with glucose as a standard, and the total protein content by the Lowry method with bovine serum albumin (BSA) as a standard [21]. The monosaccharide composition was analyzed using the PMP-HPLC method as described previously [23,24,25]. In brief, 2 mg of the EPS-LM sample was hydrolyzed with 2 M TFA at 110 °C for 4 h under a nitrogen atmosphere, dried under vacuum, and redissolved in deionized water. The solution was derivatized with 0.5 M PMP in methanol and 0.3 M NaOH, incubated at 70 °C for 30 min, and then neutralized with 0.3 M HCl. Following chloroform extraction, the aqueous layer was analyzed by high-performance liquid chromatography (HPLC) on an Agilent Zorbax Eclipse XDB-C18 column (150 mm × 4.6 mm) using a mobile phase consisting of 0.05 M phosphate buffer with 15% acetonitrile (solvent A) and 40% acetonitrile (solvent B). The individual monosaccharide peaks were identified and quantified through calibration with monosaccharide standards (Sigma, St. Louis, MO, USA).

#### 2.2.2. Determination of Molecular Weight

The MW of EPS-LM was determined by high-performance gel permeation chromatography (HPGPC) as previously reported with slight modifications [23]. The system consisted of an Agilent 1260 Infinity HPLC with a G1362A refractive index detector (Agilent Technologies, Santa Clara, CA, USA) and a Waters Ultrahydrogel Linear column (7.8 mm × 300 mm; Waters Co., Milford, MA, USA). The detector and column were both maintained at 40 °C. The mobile phase consisted of 0.1 M NaNO_3_, operated at a flow rate of 1 mL/min. Sample solutions were filtered through a 0.22 μm membrane filter prior to injection, and 20 μL of each sample was injected into the chromatographic system. Polyethylene glycol (PEG) standards (MW 4.30 × 10^2^–3.26 × 10^5^ Da) were used for calibration.

#### 2.2.3. FT-IR Analysis

Infrared (IR) spectroscopic analysis was conducted at room temperature using an Avatar 360 Fourier-transform infrared (FT-IR) spectrometer (Thermo Nicolet, Cambridge, UK). The spectra were recorded in the range of 4000–500 cm^−1^ with a resolution of 4 cm^−1^. For analysis, the solid sample was mixed with potassium bromide (KBr) powder, ground, and pressed into a pellet [21].

#### 2.2.4. Amino Acid Analysis

The total amino acid (TAA) composition of EPS-LM was determined using an automatic amino acid analyzer (LA8080, Hitachi, Japan) following a reported method [26] with minor modifications. A 100 mg sample was hydrolyzed in sealed glass tubes with 6 mol/L HCl at 110 °C for 23 h. The hydrolysate was diluted to 10 mL with deionized water, filtered through two layers of filter paper, and 1.0 mL of the filtrate was transferred to a 50 mL beaker, where it was dried using an electric platen. The dried material was redissolved in 1.0 mL of 0.02 mol/L HCl for analysis. Amino acids were separated using cation exchange chromatography and quantified via ninhydrin post-column derivatization, with detection at 570 nm and 440 nm. The amino acid concentrations were calculated directly by the instrument, based on their absorption intensities in reference to the amino acid standards.

#### 2.2.5. UV–Vis and Fluorescence Spectral Analysis

The UV-Vis absorbance spectra of EPS-LM were recorded using a Varian Cary 4000 UV-Vis Spectrophotometer (Varian Co., Palo Alto, CA, USA). Samples were prepared at four concentrations, 1.0, 0.5, 0.25, and 0.125 mg/mL in deionized (DI) water, with DI water serving as a control. Absorbance was measured over a wavelength range of 200–800 nm using a 1 cm path length quartz cuvette at room temperature. The same sample concentrations were used for fluorescence analysis, and the fluorescence spectrum was recorded using a fluorescence spectrophotometer (Agilent Technologies). The excitation wavelength was set to 280 nm, while the emission spectrum was scanned between 300 and 450 nm. Both the excitation and emission slit widths were adjusted to 5 nm to ensure optimal resolution and sensitivity.

#### 2.2.6. Circular Dichroism Analysis

Circular dichroism (CD) was measured using a J-1500 CD spectropolarimeter (JASCO Co., Tokyo, Japan) at room temperature with a 0.1 cm path length cuvette. The EPS-LM sample was prepared in PBS, and spectra were collected at a scan speed of 100 nm/min, with a digital integration time of 1 s and a bandwidth of 4 nm. For far-UV analysis, measurements were performed over the range of 260 to 190 nm with 1 nm step increments, and at least two scans were averaged. Secondary structural analysis was conducted using the JWSSE-513 program, which applies the classical least squares (CLS) method and incorporates the reference spectra [22].

#### 2.2.7. Endotoxin Detection

The endotoxin level of EPS-LM was determined using a chromogenic Limulus amebocyte lysate (LAL) assay kit (Beyotime, Shanghai, China) according to the manufacturer’s instructions. EPS-LM solutions were prepared in endotoxin-free water, and endotoxin-free consumables were used throughout. An endotoxin standard provided with the kit was used to construct a calibration curve over the range of 0.1–1.0 EU/mL, and sample absorbance at 545 nm was converted to endotoxin concentration (EU/mL) and normalized to EPS-LM mass (EU/mg).

### 2.3. In Vitro Antioxidant Activity Assays

#### 2.3.1. DPPH Radical-Scavenging Assay

The 2,2-diphenyl-1-picrylhydrazyl (DPPH) radical scavenging activity of the samples was determined with slight modifications of a reported method [27]. EPS-LM samples were dissolved in deionized water to obtain concentrations ranging from 0.25 to 3 mg/mL. A 50 μg/mL DPPH working solution in ethanol was freshly prepared, and 3 mL of the DPPH solution was mixed with 1 mL of the diluted EPS-LM solution in the dark at room temperature. According to the time-resolved conceptual approach of Marullo et al. [28], the reaction mixtures were first incubated for 30 min, which was considered the initial time point (defined as 0 h), and then further incubated to monitor the long-acting radical scavenging behavior of the macromolecular EPS-LM at 6, 12 and 24 h. Vitamin C (VC, 30 μg/mL; 0.17 mM) was included as a small-molecule, fast-acting antioxidant reference control. At the indicated time points, the absorbance (A) of the supernatant was measured at 517 nm using a microplate reader (Thermo Scientific, Waltham, MA, USA). A mixture of the DPPH working solution and deionized water served as the control. The DPPH radical scavenging activity (%) was calculated as (1-Asample/Acontrol) × 100, where Asample and Acontrol are the absorbances of EPS-LM sample and the control, respectively.

#### 2.3.2. ABTS Radical-Scavenging Assay

The 2,2-azino-bis(3-ethylbenzothiazoline-6-sulfonic acid) (ABTS) radical-scavenging activity of the samples was determined according to a reported method [29] with slight modifications. A stock ABTS solution was prepared by dissolving 0.007 g of K_2_S_2_O_8_ and 0.0406 g of ABTS in 10 mL of distilled water. The solution was kept in the dark at room temperature for 16 h to generate the ABTS radical cation. Prior to use, the ABTS stock solution was diluted with distilled water until the absorbance at 734 nm reached 0.70 ± 0.02, and the resulting solution was used as the ABTS working solution. To evaluate ABTS radical scavenging activity, 3.6 mL of ABTS working solution was mixed with 0.4 mL of diluted EPS-LM and incubated at room temperature for 10 min (defined as 0 h). VC, 0.17 mM was used as a small-molecule, fast-acting antioxidant reference. The control was prepared in the same way as the sample mixtures, except that distilled water was used instead of the sample. Absorbance at 734 nm was measured at 0, 6, 12, and 24 h using a microplate reader. The ABTS radical-scavenging activity (%) was calculated as (1 − Asample/Acontrol) × 100, where Asample and Acontrol are the absorbances of the sample and the control, respectively.

### 2.4. Assessment of EPS-LM Bioactivities

#### 2.4.1. Caco-2 Intestinal Epithelial Cell Culture

The Caco-2 human colonic adenocarcinoma cell line and Eagle’s Minimum Essential Medium (EMEM) were purchased from the American Type Culture Collection (ATCC, Manassas, VA, USA). Cells were cultured in EMEM supplemented with 20% fetal bovine serum (FBS), 100 U/mL penicillin, and 100 μg/mL streptomycin in a humidified incubator at 37 °C with 5% CO_2_. The culture medium was refreshed every two days until the cells reached 80% confluence. Cells were then digested with 0.25% trypsin at 37 °C for 3 min and seeded into 96, 24, 12, or 6 well culture plates for further experiments. All reagents for cell culture, including Hanks’ Balanced Salt Solution (HBSS), trypsin (0.25%), antibiotic-antimycotic solution (penicillin (100 U/mL) and streptomycin (100 μg/mL)), and FBS, were obtained from Gibco Biotechnology Co., Ltd. (Grand Island, NY, USA).

#### 2.4.2. Reagents

Hydrogen peroxide (H_2_O_2_), 3-(4,5-Dimethylthiazol-2-yl)-5-(3-carboxymethoxyphenyl)-2-(4-sulfophenyl)-2H-tetrazolium (MTS), and 2,7-Dichlorodihydrofluorescein diacetate (DCFH-DA) were purchased from Sigma-Aldrich (St. Louis, MO, USA). Phenazine methosulfate (PMS) was purchased from Promega Corporation (Madison, WI, USA). Fluorescein sodium was purchased from Shanghai Reagent (Shanghai, China). Glutathione peroxidase (GSH-Px), catalase (CAT), and total superoxide dismutase (T-SOD) assay kits were purchased from Nanjing Jiancheng Bioengineering Institute (Nanjing, China). Transwell permeable supports (0.4 µm pore size) and cell culture plates were purchased from SPL Life Sciences Co., Ltd. (Gyeonggi-do, Republic of Korea). The Bicinchoninic acid (BCA) protein assay kit was purchased from Thermo Fisher Scientific (Rockford, IL, USA). Calcein acetoxymethyl ester/propidium iodide (Calcein-AM/PI) Live/Dead Cell Double Staining Kit was purchased from Servicebio (Wuhan, China). All other chemicals and reagents were of analytical grade.

#### 2.4.3. Cell Viability Assay

The cell viability was assessed using the MTS assay [22]. Caco-2 cells in the logarithmic growth phase were seeded into 96-well plates at a density of 1 × 10^5^ cells/mL (100 μL/well) and incubated for 24 h. To select the suitable concentration of H_2_O_2_ for the ROS-induced cell damage and the protective effect of EPS-LM, preliminary tests were performed by treating the cells at various concentrations of H_2_O_2_ (0–700 μM) for 24 h. The results showed (Supplementary Figure S3) that cell viability started to decrease significantly when H_2_O_2_ concentrations exceeded 500 µM and at 550 µM, the cell viability reduced by 50% compared to the control. Therefore, 550 µM H_2_O_2_ was selected to induce oxidative damage in this study. For the protective effect of EPS-LM against H_2_O_2_-induced cytotoxicity, cells were simultaneously exposed to 550 μM H_2_O_2_ and EPS-LM at concentrations of 50, 100, 200, and 400 μg/mL for 24 h. After the respective treatments, the culture medium in the 96-well plates was removed, and 45 μL of MTS solution mixed with PMS was added to each well. The plates were incubated at 37 °C for 2 h, and the optical density (OD) at 492 nm was measured using a microplate reader. In parallel, cell-free wells containing culture medium, MTS/PMS and EPS-LM (cell-free) were processed under identical conditions. As shown in Supplementary Figure S4, EPS-LM cause no significant increase in the absorbance at 492 nm, indicating its negligible interference with the MTS readout.

#### 2.4.4. Cell Viability Staining Assay

To further elucidate the protective effects of EPS-LM (50 and 200 μg/mL) on Caco-2 cells following exposure to 550 μM H_2_O_2_, the cells were stained using the Calcein-AM/PI Double Stain Kit. The staining process involved incubating the cells with calcein AM and PI for 30 min, following the manufacturer’s protocol. Fluorescent images were subsequently captured using an inverted fluorescence microscope (Ti2-E, Nikon Co., Tokyo, Japan).

#### 2.4.5. Intracellular ROS Assay

Intracellular ROS levels were quantified using the method of [21]. DCFH-DA is a fluorogenic probe for general intracellular ROS. Cells were plated in 96-well plates at a concentration of 1 × 10^5^ cells/mL, following the procedures described in Section 2.4.1. Inside the cells, DCFH-DA is deacetylated by esterases into a non-fluorescent compound, which is then oxidized by ROS to form 2′,7′-dichlorofluorescein (DCF). The fluorescence of DCF was measured using a microplate reader with excitation at 485 nm and emission at 527 nm.

#### 2.4.6. Measurement of Antioxidant Enzyme Activities

Caco-2 cells were harvested and lysed with an ultrasonic cell disruptor while kept in cold water to maintain a low temperature and prevent protein degradation, and the resulting supernatants were collected for subsequent analysis. The protein concentration was determined using the BCA assay, following the protocol provided by the manufacturer. Total activities of T-SOD, CAT and GSH-Px in whole cell lysates were measured using commercial kits (Nanjing Jiancheng, Nanjing, China) according to the manufacturer’s instructions. Enzyme activities were first expressed as units per mg of protein (U/mg protein) and then normalized to the activity of the untreated control group, which was set to 100%. Changes in T-SOD, CAT and GSH-Px activities are presented as percentage of control and normalized to total protein.

#### 2.4.7. TEER Measurement

The TEER assay was conducted to evaluate the TJ integrity of the Caco-2 monolayer [30]. Caco-2 cells were seeded at a density of 1 × 10^5^ cells/mL onto Transwell membranes (polyester, 12 mm diameter, 0.4 μm pore size). A well without cells served as the blank control. The medium in the apical (AP) and basolateral (BL) chambers (0.2 mL and 0.6 mL, respectively) was replaced every 3 days. After 21 days of culture on Transwell inserts, the Caco-2 cells spontaneously differentiated into polarized monolayers. For protection studies, monolayers were allocated to the following groups for 24 h: (i) Control (no treatment); (ii) H_2_O_2_ Model (550 μM H_2_O_2_ alone); and (iii) H_2_O_2_ + EPS-LM co-treatment (550 μM H_2_O_2_ with EPS-LM at 50 or 200 μg/mL). Baseline TEER (TEER0) was recorded immediately before initiating the 24 h treatment. TEER (Ω·cm^2^) was measured once again immediately after completing the 24 h treatment using a Millicell Electrical Resistance System (ERS-2, Millipore-Sigma, Burlington, MA, USA) following blank correction, and calculated as (Rm − Ri) × A, where Rm is the measured trans-epithelial resistance, Ri is the intrinsic resistance of cell-free medium, and A is the membrane surface area (cm^2^). The change in resistance was defined as ΔTEER (Ω·cm^2^) = TEER 24h − TEER0 (TEER0 and TEET 24h: TEER values measured at 0 and 24 h after the treatment respectively); negative values indicate a decrease from baseline.

#### 2.4.8. Cell Permeability Assay

The permeability assay using sodium fluorescein was adapted from previous studies [31,32], with permeability reported as BL concentration (μg/mL) under sink conditions. After TEER evaluation, monolayers were gently washed three times with Hank’s Balanced Salt Solution (HBSS, pH 7.4). During the final wash, cells were incubated at 37 °C for 30 min to allow equilibration. Then, 200 μL of sodium fluorescein solution (250 μg/mL) was added to the AP side, and 800 μL of HBSS was added to the BL side. BL samples (200 μL) were collected after 150 min. Absorbance was measured at 490 nm using a microplate reader, and BL concentrations (μg/mL) were determined from external calibration (R^2^ ≥ 0.999).

#### 2.4.9. RT-qPCR Analysis

Caco-2 cells were seeded into 12-well plates and incubated for 10 days to reach full differentiation. To evaluate the effects of H_2_O_2_-induced oxidative stress and the protective role of EPS-LM, the cells were treated with H_2_O_2_ and EPS-LM prior to RNA extraction. Total RNA was extracted using TRIzol Reagent (Invitrogen, Carlsbad, CA, USA) according to the supplier’s protocol. The purity and concentration of RNA were measured using a NanoDrop 2000c Spectrophotometer (Thermo Fisher Scientific, Waltham, MA, USA). Primers specific to target genes were designed using Primer-BLAST, and real-time quantitative polymerase chain reaction (RT-qPCR) was performed with the Hifair III One-Step RT-qPCR SYBR Green Kit (Yeasen, Shanghai, China). Reverse transcription (RT) was conducted at 42 °C for 10 min, followed by qPCR on an ABI QuantStudio 5 Real-time PCR system (Applied Biosystems, Foster City, CA, USA) under the following conditions: 95 °C for 5 min, then 40 cycles of 95 °C for 10 s and 60 °C for 30 s. The relative gene expression levels were calculated using the 2^−ΔΔCT^ method, with GAPDH as the reference gene [33]. The specific primer sequences used in the experiment are listed in Table 1.

#### 2.4.10. Western Blotting Analysis

Western blotting was performed to analyze the expression of tight junction and antioxidant-related proteins, following a reported protocol [21]. Caco-2 cells were seeded in 6-well plates. For antioxidant-related protein analysis, cells were cultured for 24 h to reach confluence, while for TJ protein analysis, cells were differentiated over 10 days. After treatment with H_2_O_2_ and EPS-LM for 24 h, cells were collected and washed twice with cold phosphate-buffered saline (PBS, pH 7.4). Cytoplasmic proteins were extracted using RIPA buffer (Sigma-Aldrich, USA), following the manufacturer’s instructions. Protein concentrations were quantified using the BCA kit. The primary antibodies used in this study included rabbit anti-NQO1 antibody (#T56710, Abmart, Shanghai Co., Ltd., Shanghai, China), anti-ZO-1 antibody (#GB111402, Servicebio, Wuhan, China), anti-Claudin-1 antibody (#GB12032, Servicebio, Wuhan, China), anti-NRF2 antibody (#ab62352, Abcam, Cambridge, UK), and anti-β-actin antibody (#GB15003, Servicebio) used as a loading control. For each sample, 20 µg of total protein was separated on a 10% SDS-PAGE gel, transferred onto a polyvinylidene difluoride (PVDF) membrane, and blocked with 5% skimmed milk at room temperature. The membranes were incubated overnight at 4 °C with the respective primary antibodies to allow specific binding, followed by incubation with horseradish peroxidase (HRP)-conjugated secondary antibodies to enable signal detection. Protein bands were subsequently visualized using enhanced chemiluminescence (ECL) reagents and detected through autoradiography for further analysis.

#### 2.4.11. Immunofluorescence and Confocal Microscopy Analysis

Caco-2 cells were seeded onto collagen-coated coverslips in a 12-well plate. After 10 days of culture, when the cells were fully confluent and differentiated, they were treated with H_2_O_2_ and EPS-LM for 24 h. The cells were then fixed with 4% paraformaldehyde for 10 min at room temperature and permeabilized with 0.1% Triton X-100 for 10 min, followed by three PBS washes. To block nonspecific binding, the cells were incubated with 1% BSA in PBS for 30 min. They were then incubated with primary antibodies against Claudin-1 at 4 °C for 1 h, washed three times with PBS, and incubated with FITC-conjugated secondary antibodies at room temperature for 1 h in the dark. After washing three times with PBS, the nuclei were stained with DAPI (4′,6-diamidino-2-phenylindole) for 5 min, followed by three additional PBS washes. Fluorescent images were captured using a microscope (Nikon Eclipse Ti2-E) to evaluate Claudin-1 expression and localization [21].

### 2.5. Statistical Analysis

Statistical analyses of the experimental data were performed using GraphPad Prism 10 (GraphPad Software, La Jolla, CA, USA). For each dataset, the assumptions of one-way analysis of variance (ANOVA) were assessed prior to hypothesis testing. Homogeneity of variances was evaluated using the Brown-Forsythe test. When this assumption was satisfied (*p* > 0.05), ordinary one-way ANOVA was used for multiple group comparisons, followed by Tukey’s post hoc test. Data are presented as mean ± SEM from at least three independent experiments. A *p*-value < 0.05 was considered statistically significant.

## 3. Results

### 3.1. Chemical Composition and Molecular Weight of EPS-LM

EPS-LM contained 19.3% (*w*/*w*) total carbohydrate and 28.7% (*w*/*w*) protein (Table 2), consistent with previous reports [21], demonstrating the reproducibility of EPS-LM production from Cs-HK1 mycelial fermentation. HPGPC (Supplementary Figure S1) revealed two predominant fractions with apparent molecular weights of 2.50 × 10^4^ Da (41.9% area) and 1.70 × 10^3^ Da (54.0% area) (Table 2). These size distributions were comparable to earlier findings on Cs-HK1 EPS fractions [19], which reported a broad MW range (5–200 kDa). High-molecular-weight polysaccharides have been proposed to protect the intestinal barrier by forming a hydrophilic gel-like layer that mimics intestinal mucus and may interact with membrane receptors to reinforce epithelial stability [16,34]. By contrast, the stronger antioxidant activity associated with the low-MW fraction likely reflects its higher protein content, consistent with previous observations that protein-rich, low-MW EPS fractions exhibit greater antioxidant capacity [19,35]. In addition, the endotoxin content of the EPS-LM stock solution (5 mg/mL), as determined by a chromogenic LAL assay, was ≤0.1 EU/mL (equivalent to ≤0.02 EU/mg), which is close to the lower limit of quantification of the assay, indicating negligible endotoxin contamination in the samples used for subsequent cell experiments.

### 3.2. Structural Characteristics

**Figure 1:**
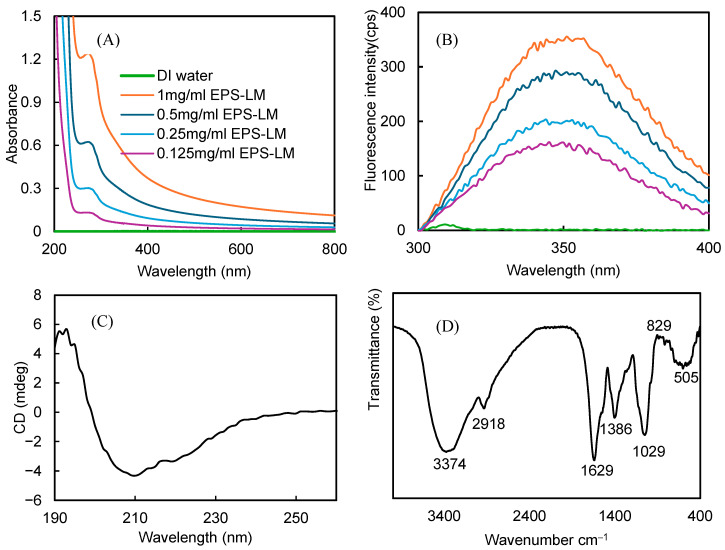
Spectroscopic characteristics of EPS-LM: (**A**) UV-Vis absorbance spectra at different concentrations; (**B**) fluorescence intensity (cps) at various concentrations; (**C**) circular dichrism (CD); (**D**) FT-IR spectrum.

As shown in Figure 1A, EPS-LM exhibited a distinct absorption band in the near-UV region (250–310 nm), which is characteristic of protein like chromophores and therefore suggests the presence of proteinaceous components in the sample. UV visible spectroscopy is a simple and direct method for detecting proteins, typically showing absorption between 250 and 310 nm [36]. Fluorescence spectroscopy is a versatile and sensitive technique for high throughput screening [37], particularly useful for rapid protein analysis [36]. As shown in Figure 1B, the fluorescence spectrum of EPS-LM (excited at 280 nm) displayed an emission band at 340–350 nm. This emission profile is consistent with that of aromatic amino acid residues (e.g., tryptophan, tyrosine, and phenylalanine) in protein-containing samples, although possible matrix effects from the EPS-LM backbone cannot be completely excluded. EPS-LM exhibited the strongest fluorescence intensity at 1 mg/mL, and the signal decreased proportionally with dilution, consistent with its concentration dependent fluorescence behavior.

CD is an effective technique for elucidating the secondary structural features of macromolecules, including proteins, polysaccharides, and glycoproteins [38,39]. In aqueous solution, the sugar chains of glycoproteins undergo dynamic conformational changes such as helix formation, folding, turning, and random coiling, which enhance molecular asymmetry [40]. As shown in Figure 1C and Table 2, the CD spectrum of EPS-LM exhibited a positive Cotton effect around 195 nm and a negative Cotton effect near 210 nm. Analysis of the CD data revealed that the secondary structure of EPS-LM was primarily composed of β-sheets (49.8%), along with random coils (30.8%) and α-helices (19.3%). The predominance of β-sheets suggested a highly ordered and stable conformation, which may enhance molecular integrity and facilitate interactions with polysaccharides, contributing to protein stability and functionality [41,42,43]. The considerable proportion of random coils indicated structural flexibility, supporting dynamic interactions with polysaccharides and contributing to functional versatility [44].

The FT-IR spectrum (Figure 1D) provided further structural information on EPS-LM. The broad absorption band at 3374 cm^−1^ was attributed to O-H stretching vibrations, indicative of hydroxyl groups commonly present in polysaccharides and proteins. The peak at 2918 cm^−1^ corresponded to C-H stretching vibrations originating from aliphatic groups or C-H bonds within polysaccharide chains [21]. The peaks at 1629 cm^−1^ and 1386 cm^−1^ were assigned to the amide I and amide II bands, respectively, confirming the presence of proteins. Specifically, the amide II band at 1386 cm^−1^ likely arose from N-H bending and C-N stretching vibrations, with potential shifts influenced by hydrogen bonding or interactions between proteins and polysaccharides [22]. Additionally, the peak at 1029 cm^−1^ was associated with C-O-C and C-OH stretching vibrations, indicating the presence of polysaccharide moieties [23].

The ^1^H-NMR spectrum (Supplementary Figure S2) displayed signals in the range of 4.4–5.5 ppm, characteristic of the anomeric protons in polysaccharides, confirming their presence [45]. Signals between 3.0 and 4.4 ppm were attributed to the side-chain hydrogens of amino acids such as glutamine, glutamic acid, and alanine, while those between 2.5 and 3.0 ppm also originated from side-chain hydrogens, specifically from glutamine and arginine [46,47,48]. Additionally, signals in the range of 1.2–1.5 ppm corresponded to the methyl groups of branched-chain amino acids, including isoleucine and valine, whereas signals between 0.8 and 1.0 ppm were characteristic of the methyl groups in aliphatic amino acids such as leucine and isoleucine [22,49]. These ^1^H-NMR findings, together with the FT-IR results, further supported the presence of both protein and polysaccharide components in EPS-LM.

### 3.3. Monosaccharide and Amino Acid Composition

As shown in Table 2, the monosaccharide composition of EPS-LM revealed mannose as the dominant sugar with a molar ratio of 2.127, followed by galactose (1), glucose (0.4946), and galactosamine (0.2688). These findings indicated that EPS-LM is a mannose-rich heteropolysaccharide. The overall monosaccharide profile was consistent with previous reports on EPS derived from *C. sinensis* Cs-HK1 [21], which also contained mannose, galactose, and glucose as the main carbohydrate constituents. The amino acid composition of EPS-LM is presented in Table 3. The total amino acid content (TAA) was 210.4 mg/g, comprising 65.0 mg/g of essential amino acids (EAAs) and 145.4 mg/g of non-essential amino acids (NAAs). Aspartic acid (43.7 mg/g), glutamic acid (22.2 mg/g), and glycine (21.8 mg/g) were the predominant NAAs, whereas threonine (19.3 mg/g) and lysine (12.4 mg/g) were the most abundant EAAs. The amino-acid pattern of EPS-LM was comparable to that reported for the glycopeptide Cs-GP1 isolated from Cs-HK1 [35]. These compositional data suggest that EPS-LM contains both polysaccharide and protein components, consistent with its characterization as a protein–polysaccharide complex.

### 3.4. Antioxidant Activities

**Figure 2:**
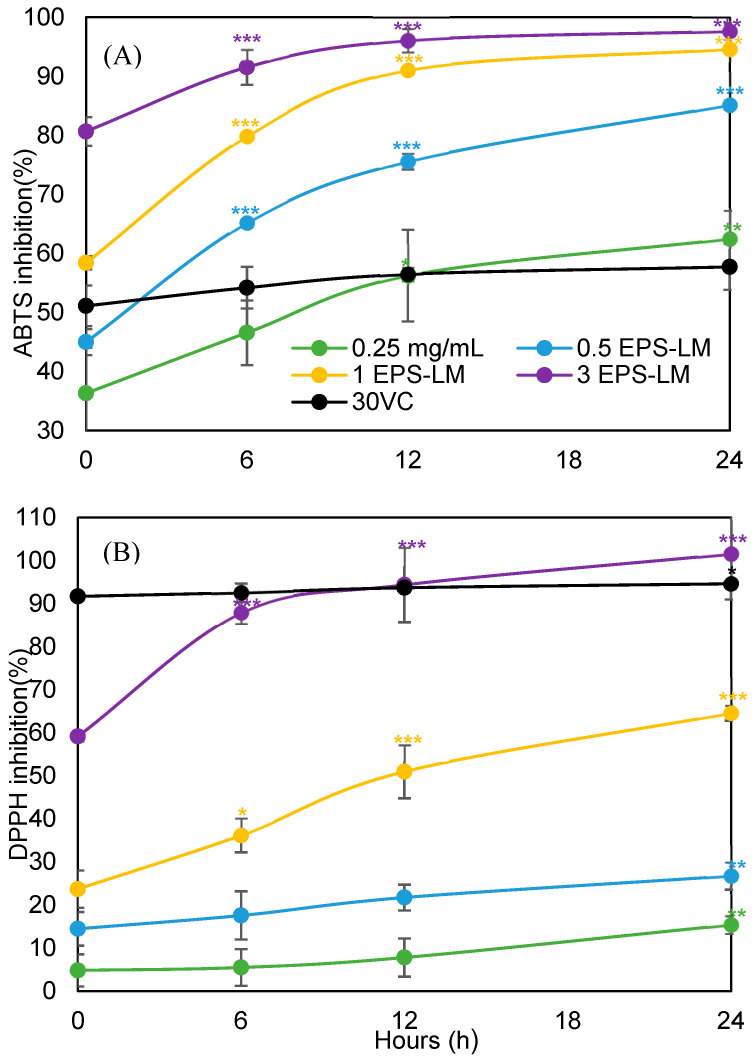
Radical-scavenging activity of EPS-LM at different concentrations (mg/mL) and incubation times: (**A**) ABTS assay and (**B**) DPPH assay, with vitamin C (30 μg/mL) as an antioxidant reference. Data are presented as mean ± SEM (*n* = 6). * *p* < 0.05, ** *p* < 0.01, *** *p* < 0.001 vs. 0 h (for each concentration).

Consistent with its small molecular size and high reactivity, VC showed high and almost time independent radical scavenging activity in both assays, maintaining about 50–60% inhibition in the ABTS assay and >90% in the DPPH assay from 0 to 24 h, indicating a rapid reaction completed within the usual incubation period. By contrast, EPS-LM showed a clear time and concentration dependent increase in activity (Figure 2). In the ABTS assay, inhibition at 1–3 mg/mL rose from about 59–81% at 0 h to 94% at 24 h, and at 0.25–0.5 mg/mL from about 32–45% to 60–85%. Consistently, the ABTS IC_50_ of EPS-LM almost halved from 0.689 mg/mL at 0 h to about 0.30 mg/mL at 24 h. In the DPPH assay, EPS-LM at 3 mg/mL increased from about 60% inhibition at 0 h to nearly 100% at 24 h, and at 1 mg/mL from about 26% to over 60%. In line with this, the DPPH IC_50_ decreased from 2.023 mg/mL at 0 h to 0.807 mg/mL at 24 h (Table 4). Overall, EPS-LM did not match the very rapid, high inhibition of VC, but its scavenging capacity increased markedly over time and eventually reached a high and sustained level after 24 h, indicating a slow acting, long lasting antioxidant effect. These contrasting time dependent behaviors were the rationale for extending the observation window to 24 h in the present study, to distinguish the rapid, almost time independent response of the small molecule antioxidant VC from the slow acting, time dependent antioxidant response of the macromolecular EPS LM, in line with previous reports on polysaccharide-based antioxidant systems [28].

### 3.5. Effects of EPS-LM on Caco-2 Cell Viability

**Figure 3:**
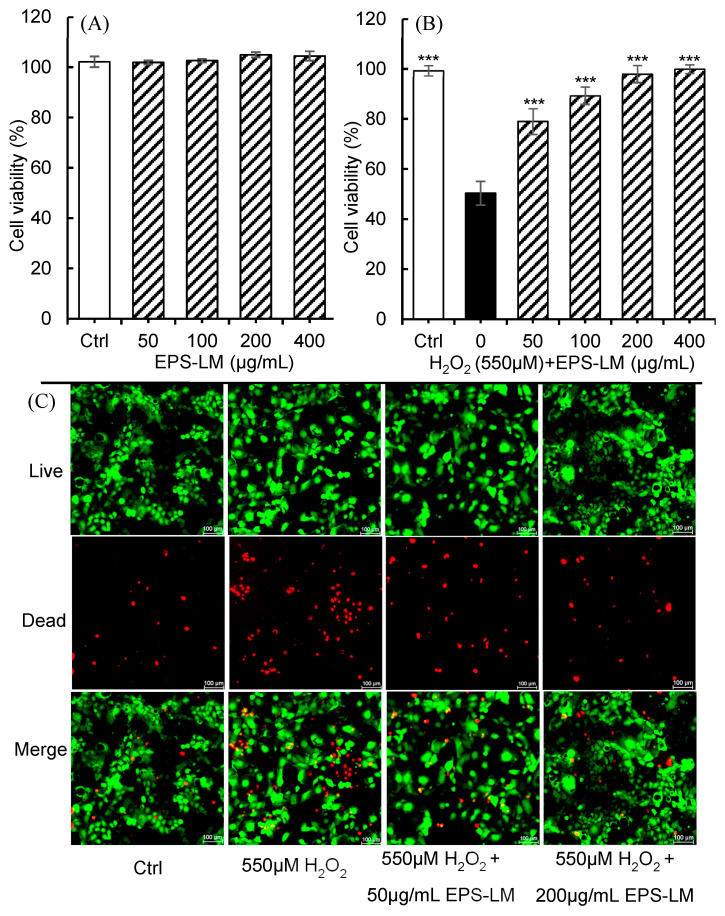
Impact of H_2_O_2_ and EPS-LM on Caco-2 cell viability: (**A**) treatment with EPS-LM alone at 0, 50, 100, 200, and 400 µg/mL; (**B**) treatment with 550 µM H_2_O_2_ plus EPS-LM at 50, 100, 200, and 400 µg/mL; (**C**) confocal microscopy images of cells stained with calcein AM (green) for viable cells and propidium iodide (PI, red) for dead cells in control, 550 µM H_2_O_2_, and 550 µM H_2_O_2_ plus 50 or 200 µg/mL EPS-LM. Data are presented as mean ± SEM (*n* = 6). *** *p* < 0.001 vs. H_2_O_2_.

Figure 3A shows that EPS-LM was non-toxic, with no significant change in Caco-2 cell viability across 0–400 µg/mL, and cell-free MTS controls excluded any direct interference of EPS-LM with the assay readout (Supplementary Figure S4). In Figure 3B, EPS-LM demonstrated a dose-dependent ability to preserve cell viability under H_2_O_2_ challenge, with the highest concentration (400 µg/mL) almost restoring viability to control levels. This protective effect was consistently observed across a broader range of H_2_O_2_ concentrations (350–700 µM), as shown in Supplementary Figure S5. Figure 3C presents fluorescence microscopy images in which cells exposed to 550 μM H_2_O_2_ showed intense red fluorescence (PI, dead cells) and reduced green fluorescence (Calcein-AM, viable cells). Treatment with 50 or 200 µg/mL EPS-LM markedly decreased red fluorescence and increased green fluorescence in a dose-dependent manner, consistent with the MTS assay results.

### 3.6. Effects of EPS-LM on Antioxidant Responses in Caco-2 Cells

#### 3.6.1. EPS-LM Reduces ROS Levels and Restores Antioxidant Enzyme Activities

**Figure 4:**
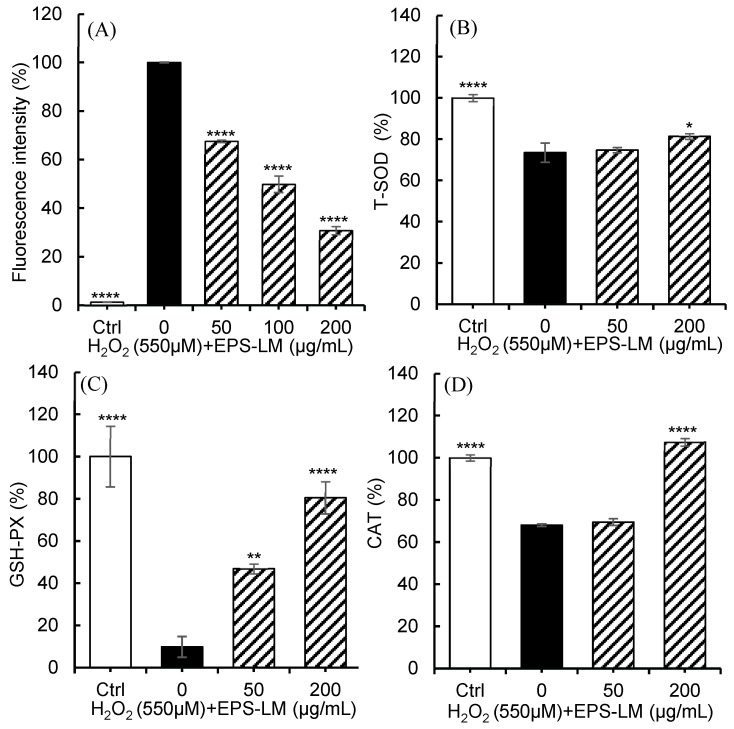
Effects of EPS-LM on intracellular ROS levels and antioxidant enzyme activities in Caco-2 cells exposed to H_2_O_2_. (**A**) ROS levels following exposure to H_2_O_2_ with or without EPS-LM treatment; (**B**) T-SOD; (**C**) GSH-Px; and (**D**) CAT. Data are presented as mean ± SEM (*n* = 6). * *p* < 0.05, ** *p* < 0.01, **** *p* < 0.0001 vs. 550 µM H_2_O_2_.

Exposure of Caco-2 cells to 550 µM H_2_O_2_ significantly increased intracellular ROS levels (Figure 4A), confirming the induction of oxidative stress. Treatment with EPS-LM reduced ROS accumulation in a concentration-dependent manner, with the maximal reduction observed at 200 µg/mL. The activities of key antioxidant enzymes were markedly affected by H_2_O_2_ exposure and subsequently restored by EPS-LM. As shown in Figure 4B–D, treatment with 550 µM H_2_O_2_ significantly decreased the activities of T-SOD, CAT and GSH-Px, whereas co-treatment with EPS-LM (50–200 µg/mL) dose-dependently recovered these enzyme activities. The highest restoration was observed at 200 µg/mL EPS-LM, consistent with its effect on ROS reduction. These results indicate that EPS-LM effectively mitigated H_2_O_2_-induced oxidative stress by decreasing intracellular ROS levels and promoting the recovery of antioxidant enzyme activities.

#### 3.6.2. EPS-LM Modulates NRF2-NQO1 Signaling Under Oxidative Stress

**Figure 5:**
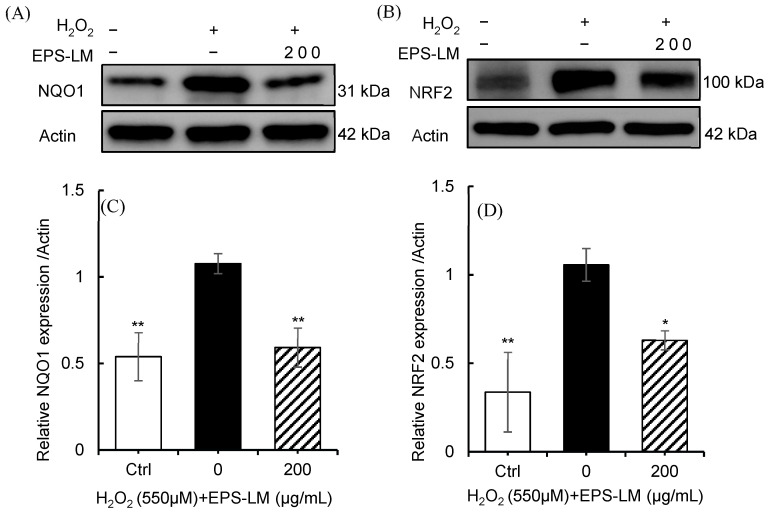
Effects of EPS-LM on NRF2 and NQO1 expression in Caco-2 cells under H_2_O_2_-induced oxidative stress. (**A**,**B**) Western blot analysis showing representative bands for NQO1 and NRF2 expression; (**C**,**D**) quantification of NQO1 and NRF2 expression levels. Data are presented as mean ± SEM (*n* = 3). * *p* < 0.05, ** *p* < 0.01 vs. 550 µM H_2_O_2_.

To assess oxidative burden and the involvement of the NRF2 pathway, NQO1 and total NRF2 protein levels were quantified by Western blotting and densitometry (Figure 5A–D). NQO1 limits ROS generation by catalyzing the two-electron reduction of quinones to hydroquinones, thereby preventing redox cycling and the formation of semiquinone radicals and superoxide, whereas NRF2 coordinates antioxidant defenses by activating ARE-dependent genes under oxidative stress, as previously described [6,50]. Exposure of Caco-2 cells to 550 µM H_2_O_2_ significantly increased both NQO1 and total NRF2 levels compared with the control, indicating activation of the oxidative-stress response. Treatment with EPS-LM (200 µg/mL) attenuated these H_2_O_2_-induced increases, resulting in NQO1 and NRF2 levels approaching those of the untreated control. Together with the results shown in Figure 4, these findings indicate that EPS-LM alleviated oxidative stress by lowering intracellular ROS, restoring antioxidant enzyme activities, and normalizing NRF2-NQO1 signaling, thereby contributing to redox balance in Caco-2 cells.

### 3.7. Protective Effects of EPS-LM on Epithelial Barrier Function in Caco-2 Cell Culture

#### 3.7.1. EPS-LM Alleviates H_2_O_2_-Induced Epithelial Barrier Dysfunction

**Figure 6:**
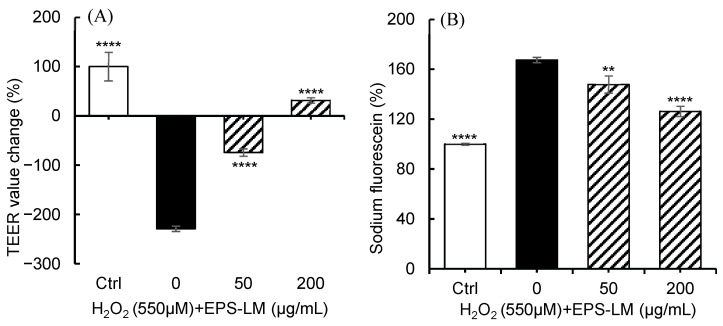
Effects of EPS-LM on H_2_O_2_-induced changes in transepithelial electrical resistance (TEER) and sodium fluorescein permeability in Caco-2 cells. (**A**) TEER values after 24 h (% of control); (**B**) basolateral sodium fluorescein levels (% of control) at 150 min. Data are presented as mean ± SEM (*n* = 6). ** *p* < 0.01, **** *p* < 0.0001 vs. control.

The integrity of the intestinal barrier was assessed using two complementary assays: TEER and sodium fluorescein permeability [51]. TEER reflects the electrical resistance across a cell monolayer and serves as an indicator of TJ integrity, whereas sodium fluorescein measures paracellular permeability, providing a sensitive evaluation of early barrier disruption [52]. Together, these methods provide a comprehensive assessment of intestinal barrier function by combining electrical resistance and fluorescence-based permeability measurements. As shown in Figure 6, exposure of Caco-2 cells to 550 µM H_2_O_2_ for 24 h resulted in marked barrier dysfunction, evidenced by a 60 Ω·cm^2^ decrease in TEER relative to baseline (TEER_0_) and a corresponding increase in basolateral sodium fluorescein concentration to 2.58 µg/mL. Co-treatment with 50 µg/mL EPS-LM mitigated these changes, showing a smaller TEER decrease (−20 Ω·cm^2^) and a lower basolateral fluorescein level (2.36 µg/mL), indicating a protective trend at the lower dose. At 200 µg/mL, EPS-LM exerted a pronounced protective effect, with TEER values approaching or exceeding initial levels and fluorescein permeability significantly reduced to 1.90 µg/mL. These findings demonstrate that EPS-LM attenuated H_2_O_2_-induced decreases in TEER and increases in paracellular permeability in a dose-dependent manner, supporting its role in maintaining tight-junction integrity and epithelial barrier function under oxidative stress.

#### 3.7.2. EPS-LM Restores Tight-Junction Components Under Oxidative Stress

**Figure 7:**
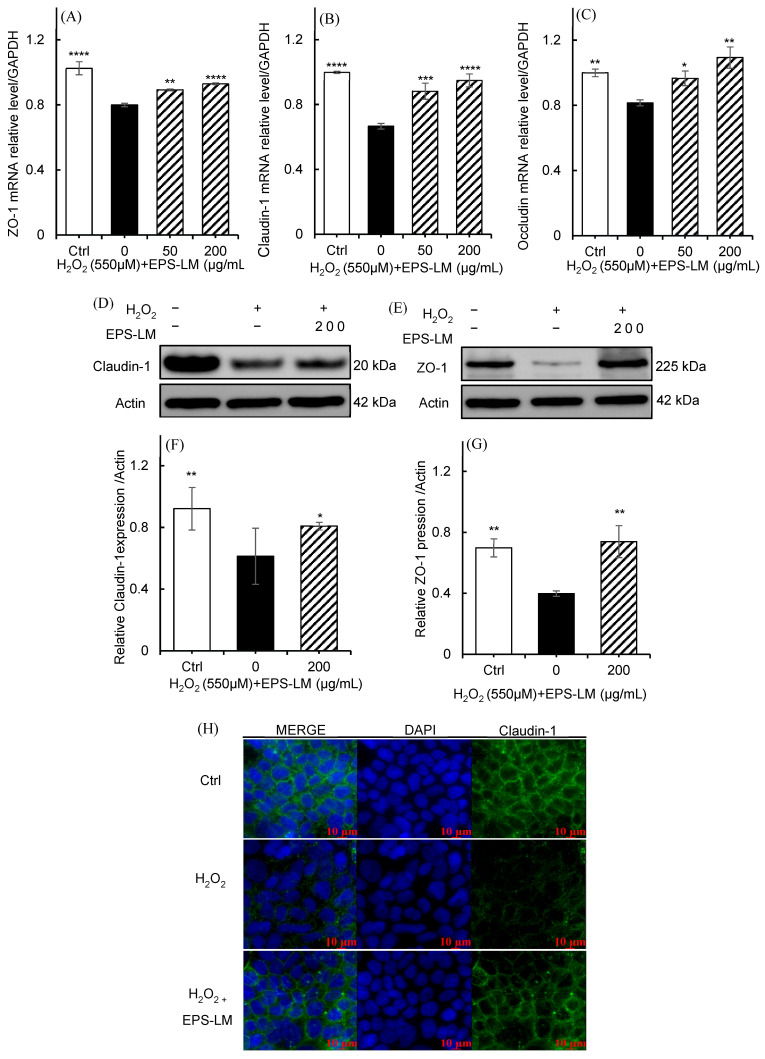
Effects of EPS-LM on tight-junction proteins in Caco-2 cells under H_2_O_2_-induced oxidative stress. (**A**–**C**) mRNA expression levels of tight-junction proteins ZO-1, Claudin-1, and Occludin in Caco-2 cells; (**D**,**E**) protein expression of Claudin-1 and ZO-1 determined by Western blotting; (**F**,**G**) quantification of Claudin-1 and ZO-1 protein levels normalized to β-actin; (**H**) Immunofluorescence staining of claudin-1 in Caco-2 cells. Data are presented as mean ± SEM (*n* = 3). * *p* < 0.05, ** *p* < 0.01, *** *p* < 0.001, **** *p* < 0.0001 vs. 550 µM H_2_O_2_.

As shown in Figure 7A–C, exposure of Caco-2 cells to 550 µM H_2_O_2_ significantly decreased the mRNA expression of the TJ genes ZO-1, Claudin-1, and Occludin, indicating oxidative stress-induced disruption of barrier-related transcripts. Treatment with 50 µg/mL EPS-LM partially restored the mRNA levels of these TJ components, and 200 µg/mL EPS-LM further enhanced their expression, showing a clear dose-dependent improvement. Consistent with the transcriptional findings, Western blot analysis and densitometric quantification (Figure 7D–G) demonstrated that EPS-LM remarkably increased Claudin-1 and ZO-1 protein levels after H_2_O_2_ exposure, with the greatest recovery observed at 200 µg/mL. Immunofluorescence imaging of Claudin-1 (Figure 7H) further confirmed the protective effect of EPS-LM, showing restoration of continuous junctional localization along cell borders. Together, these results indicate that EPS-LM preserved tight-junction gene and protein expression in a dose-dependent manner, contributing to the maintenance of epithelial barrier integrity under oxidative stress.

## 4. Discussion

Polysaccharides rich in mannose are widely recognized for their pronounced antioxidant activities. Mannose residues enhance radical-scavenging and reducing capacity and stimulate antioxidant enzyme expression [53,54,55]. Furthermore, mannose plays a role in stabilizing polysaccharide structures, such as in xanthan, where the terminal mannose reinforces the double helix [56]. The coexistence of galactose and glucose in EPS-LM may further modulate its biological effects. Galactose has been linked to macrophage activation and immunostimulation [57], whereas glucose residues confer metabolic flexibility and redox buffering capacity [58]. Thus, the heterogeneous monosaccharide composition of EPS-LM may provide complementary biochemical roles that enhance both structural integrity and functional antioxidant potential and is consistent with the general consensus that specific sugar residues contribute to the overall bioactivity of the biopolymer, although the precise structure activity relationships remain to be elucidated [54].

In addition to its sugar composition, the amino acid profile of EPS-LM may also contribute to its observed bioactivity. The overall amino acid composition of EPS-LM shows a noticeable resemblance to that reported for Cs-GP1, a bioactive glycopeptide isolated from Cs-HK1. Cs-GP1 is rich in glutamic acid, aspartic acid, and glycine and has demonstrated significant antioxidant and cytoprotective properties, effectively mitigating oxidative damage and preserving cell viability under stress conditions [35]. In addition, lysine and threonine in EPS-LM may provide complementary physiological functions, as lysine is involved in collagen formation and mineral absorption, whereas threonine supports mucin synthesis, which is essential for intestinal surface protection [59,60]. These observations suggest that the coexistence of polysaccharide and amino acid components within EPS-LM may confer complementary antioxidant and intestinal barrier protective capacities, which may provide a mechanistic basis for the potent antioxidant and cytoprotective effects observed in subsequent experiments.

Building on these compositional insights, the antioxidant assays revealed a gradual time dependent scavenging behavior of EPS-LM within the incubation period used, suggesting that effective neutralization of free radicals may require prolonged molecular interactions between EPS-LM and the reactive species. Similar gradual and time-dependent scavenging behaviors have been reported for other polysaccharide-based antioxidant systems [28], although the precise structural determinants and mechanisms underlying this behavior remain to be clarified. Taken together, these findings indicate that the gradual behavior of EPS-LM is not abnormal but rather a common characteristic of high-molecular weight polysaccharide antioxidants, milder but more sustainable, in contrast to the rapid, diffusion-controlled reactions typical of small-molecule standards such as vitamin C.

Excessive accumulation of ROS induces oxidative damage and cell death, contributing to a variety of pathological processes [61]. Consistent with the observed decreases in intracellular ROS levels and the restoration of antioxidant enzyme activities shown in Figure 4, EPS-LM effectively mitigated H_2_O_2_-induced oxidative stress through restoration of key antioxidant enzymes. SOD catalyzes the conversion of superoxide anions into hydrogen peroxide and molecular oxygen, thereby preventing superoxide accumulation and initiating ROS detoxification [62]. CAT subsequently decomposes hydrogen peroxide into water and oxygen [63], while GSH-Px reduces hydrogen peroxide and lipid hydroperoxides, protecting membrane lipids and preserving cellular integrity [64]. The significant recovery of these enzymatic antioxidant activities in EPS-LM treated cells suggests that EPS-LM supports the enzymatic defense network responsible for ROS clearance. Taken together with the DPPH and ABTS results, these findings suggest that EPS-LM exerts its antioxidant effects through a combination of direct radical scavenging and modulation of endogenous antioxidant defenses, rather than through a single mechanism alone.

In addition to direct enzymatic restoration, EPS-LM treatment tended to restore the NRF2-NQO1 signaling axis, which serves as a key cellular regulator of oxidative defenses. NQO1 limits ROS generation by catalyzing the two-electron reduction of quinones to hydroquinones, thereby preventing redox cycling and the formation of semiquinone radicals and superoxide. In parallel, NRF2 coordinates antioxidant defenses by activating ARE-dependent genes upon KEAP1 cysteine modification under oxidative stress [6,50]. NRF2 activation promotes the transcription of downstream antioxidant and phase II detoxification enzymes, including NQO1 [65], thereby reinforcing the intracellular antioxidant system. In the present study, H_2_O_2_ exposure increased NRF2 and NQO1 expression compared with control cells, whereas EPS-LM co-treatment reduced this stress induced upregulation toward basal levels. This response profile is consistent with the restoration of intracellular redox homeostasis rather than further stimulation of stress responsive signaling. Collectively, these findings imply a multifaceted protective mechanism of EPS-LM involving direct ROS scavenging together with modulation of endogenous antioxidant pathways and highlight EPS-LM as a redox-modulating polysaccharide that maintains oxidative homeostasis and protects cells from H_2_O_2_-induced injury.

Consistent with its antioxidant potential observed in Caco-2 cells, EPS LM substantially preserved intestinal epithelial barrier integrity under oxidative challenge. Both TEER and sodium fluorescein permeability assays demonstrated that EPS LM mitigated H_2_O_2_ induced barrier dysfunction, indicating reinforcement of TJ function. EPS-LM maintained the expression and junctional localization of Claudin-1, Occludin, and ZO-1, which are essential components of the TJ complex [9,10]. Oxidative stress is well known to trigger TJ disruption via activation of MLCK/MLC and RhoA/ROCK pathways, which increase cytoskeletal tension and cause separation of junctional complexes [11,66]. By suppressing ROS accumulation and maintaining antioxidant enzyme activities, EPS LM is likely to prevent this stress activated contractile events, thereby preserving junctional continuity. The ability of EPS-LM to limit ROS burden and to sustain tight junction proteins is compatible with a redox-dependent stabilization of the junction associated cytoskeleton under oxidative challenge.

**Figure 8:**
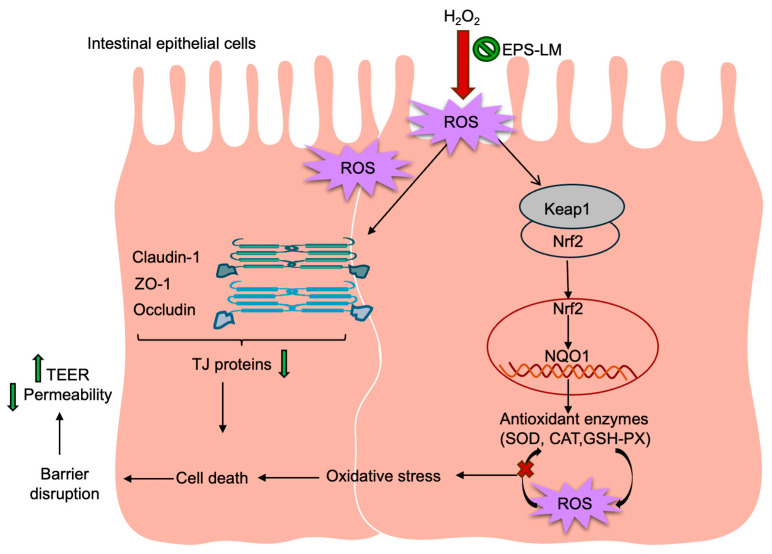
Hypothesized mechanism and signaling network for the protective effects of EPS-LM against oxidative-stress-induced epithelial barrier dysfunction in Caco-2 cell culture. The model integrates the present experimental data with previously described redox-regulated pathways in intestinal epithelial cells: (1) Exposure to H_2_O_2_ induces excessive ROS generation, damaging intestinal epithelial cells, reducing TEER, and disrupting TJ proteins (Claudin-1, ZO-1, Occludin), thereby impairing barrier integrity; (2) Accumulated ROS increase oxidative stress, leading to cellular injury and barrier dysfunction; (3) ROS promote NRF2 dissociation from KEAP1, enabling NRF2 nuclear translocation and transcriptional activation of antioxidant genes such as NQO1; (4) EPS-LM enhances SOD, CAT, and GSH-Px expression, facilitates ROS clearance, alleviates oxidative damage, and stabilizes TJ proteins, thereby protecting epithelial barrier function.

Integrating these findings, a conceptual mechanistic model is proposed (Figure 8). Under oxidative stress, excessive ROS disturb redox balance and impair TJ assembly, leading to increased epithelial permeability. EPS-LM first alleviates this oxidative burden by scavenging ROS and restoring endogenous defense systems, including SOD, CAT, and GSH-Px. By reducing oxidative tension, EPS-LM partially normalizes the KEAP1-NRF2-NQO1 signaling axis, thereby re-establishing appropriate transcriptional control of antioxidant defenses and maintaining cellular redox homeostasis. The resulting decrease in oxidative tension diminishes cytoskeletal contraction, thereby preventing TJ mislocalization and disassembly. This sequential process translates biochemical redox correction into measurable structural and functional recovery, reflected by elevated TEER, restored TJ protein networks, and reduced paracellular flux.

Comparable protective effects, in which enhanced antioxidant capacity is coupled with TJ reinforcement, have been documented for other polysaccharides and polyphenolic compounds, illustrating the generality of the mechanism proposed here. For instance, in line with this model, polysaccharide interventions in DSS-injured Caco-2 monolayers alleviated oxidative stress and improved barrier end points (TEER, paracellular permeability, TJ protein expression) [67]. Similarly, in vivo polyphenol treatment increased Occludin expression and corrected small intestinal barrier dysfunction in HFD-fed rats [66]. The pattern of antioxidant and barrier-protective effects observed for EPS-LM is in general agreement with these reports and with broader literature on natural polysaccharides and polyphenols, which commonly couple free-radical scavenging activity with preservation of tight junction integrity and epithelial barrier function under oxidative challenge [15,16].

While previous studies with polysaccharides or polyphenols mainly evaluated functional endpoints such as TEER recovery or TJ protein expression, the present work makes a new and significant contribution to this literature by combining detailed compositional and physicochemical characterization of a mannose-rich exopolysaccharide–protein complex with an integrated assessment of chemical radical-scavenging capacity, cellular redox status, antioxidant enzyme activities, NRF2-NQO1 signaling, and TJ-associated barrier function within a single experimental framework. In addition, compared with fast-acting small-molecule antioxidants such as vitamin C, which exhibit rapid and nearly time-independent radical scavenging, EPS-LM displayed a gradual, concentration and time dependent scavenging profile with progressively decreasing IC_50_ values over 0–24 h, consistent with a slow-acting and durable mode of macromolecular antioxidant action. Therefore, although EPS-LM shares the general pattern of antioxidant and barrier-protective effects reported for other natural polysaccharides and polyphenols, its mannose-rich, amino-acid-containing composition and kinetic behavior define a distinct macromolecular antioxidant profile.

## 5. Conclusions

In summary, this study demonstrates that EPS-LM attenuates H_2_O_2_-induced oxidative stress and barrier dysfunction in Caco-2 cells. EPS-LM reduced intracellular ROS levels, partially restored the activities of antioxidant enzymes, and alleviated H_2_O_2_-induced decreases in TEER and tight junction proteins, indicating antioxidant and barrier-protective effects at the epithelial level. Within the present experimental framework, EPS-LM behaved as a slow-acting, macromolecular antioxidant, showing gradual, time-dependent radical scavenging activity together with preservation of tight junction integrity. These findings are, however, derived from Caco-2 cell culture model and have not yet been extended to systems that include immune components, gut microbiota or in vivo conditions. Moreover, the possible contributions of specific monosaccharide or amino-acid residues, as well as interactions between the polysaccharide and protein moieties of EPS-LM, remain to be clarified through dedicated structure–activity studies. EPS-LM should therefore be regarded as a promising but still preliminary candidate with antioxidant and barrier-supporting properties. Future studies should include in vivo validation and co-culture models involving intestinal epithelial and immune cells (e.g., Caco-2/RAW264.7), and investigation of gut microbiota responses and structure-activity relationships.

## Figures and Tables

**Table 1:** Primer sequences of tight-junction genes used for RT-qPCR analysis.

Gene	Accession No.	Forward Primer (5′–3′)	Reverse Primer (5′–3′)
ZO-1	NM_003257.4	CTAAGGGAGCACATGGTGAAGGTAA	GTCGGGCAGAACTTGTATATGGTTT
Claudin-1	NM_021101.6	GAAGATGAGGATGGCTGTCATTGGG	GGTAAGAGGTTGTTTTTCGGGGAC
Occludin	NM_002538.3	AACTTCGCCTGTGGATGACTTCAG	TTTGACCTTCCTGCTCTTCCCTTTG
GAPDH	NM_002046.7	TCAAGAAGGTGGTGAAGCAGG	TCAAAGGTGGAGGAGTGGGT

**Table 2:** Chemical composition and structural characteristics of Cs-HK1 EPS-LM. Original GPC profiles are shown in Supplementary Figure S1. Data are presented as mean ± SEM (*n* = 3).

Total Carbohydrate (%)		Total Protein (%)	MW (Peak Area, %)
19.29 ± 0.04		28.71 ± 0.16	2.50 × 10^4^ (41.93%) 1.70 × 10^3^ (53.97%)
Protein structure (chain conformation forms)			
Random: 30.8%		α-Helix: 19.3%	β-Sheet: 49.8%
Monosaccharide composition (molar ratio)			
Mannose: 2.127	Galactose: 1	Glucose: 0.4946	Galactosamine: 0.2688

**Table 3:** Amino acid composition and contents of EPS-LM (in mg/g). Data are presented as mean ± SEM (*n* = 3).

Essential Amino Acid (EAA)		Non-Essential Amino Acid (NAA)	
Histidine	3.3 ± 0.1	Alanine	13.6 ± 0.1
Isoleucine	6.6 ± 0.1	Aspartic acid	43.7 ± 0.1
Leucine	5.7 ± 0.2	Arginine	8.4 ± 0.3
Lysine	12.4 ± 0.3	Glutamic acid	22.2 ± 0.1
Phenylalanine	7.0 ± 0.2	Glycine	21.8 ± 0.1
Threonine	19.3 ± 0.1	Serine	15.5 ± 0.1
Valine	10.7 ± 0.1	Tyrosine	3.7 ± 0.1
		Proline	16.5 ± 0.4
Total Amino Acids (TAA)		210.4 ± 1.1	
Essential Amino Acids (EAA)		65.0 ± 1.1	
Non-essential Amino Acids (NAA)		145.4 ± 0.6	
TAA (mg/g) = MW/W × V_1_/V_2_ × 10			
Where MW = amino acid MW, W = 100 mg, V_1_ = 50 mL, and V_2_ = 20 μL.			

**Table 4:** IC_50_ values (mg/mL) for the antioxidant activity of EPS-LM measured by ABTS and DPPH assays at different time points (0–24 h). Data are presented as mean ± SEM (*n* = 3). For each time point, IC_50_ values were obtained for each independent experiment by linear interpolation or extrapolation from the concentration–response curves.

Time (h)	IC_50_ (ABTS) (mg/mL) ± SEM	IC_50_ (DPPH) (mg/mL) ± SEM
0	0.689 ± 0.032	2.023 ± 0.036
6	0.296 ± 0.013	1.282 ± 0.032
12	0.34 ± 0.024	0.983 ± 0.055
24	0.305 ± 0.015	0.807 ± 0.030

## Data Availability

The original contributions presented in this study are included in the article and Supplementary Materials. Further inquiries can be directed to the corresponding author.

## Supplementary Materials

The following supporting information can be downloaded at: https://www.mdpi.com/article/10.3390/antiox14121501/s1, Figure S1: High-performance gel permeation chromatography (HPGPC) profile of EPS-LM; Figure S2: ^1^H NMR spectrum of EPS-LM; Figure S3: Preliminary dose–response analysis of H_2_O_2_-induced reduction in Caco-2 cell viability within the concentration range of 0–700 µM; Figure S4. Assessment of EPS-LM interference with the MTS assay in cell-free conditions. Cell-free wells containing culture medium, MTS/PMS and EPS-LM (50–400 µg/mL) were processed under identical conditions. Data are presented as mean ± SEM (*n* = 6). ns, not significant vs. Ctrl; Figure S5: Protective effect of EPS-LM on Caco-2 cell viability under H_2_O_2_ induced oxidative stress at concentrations ranging from 350 µM to 700 µM (A-G).
