# Supplementary material for: An Antioxidative Exopolysaccharide–Protein Complex of Cordyceps Cs-HK1 Fungus and Its Epithelial Barrier-Protective Effects in Caco-2 Cell Culture
Source: Antioxidants (Basel). 2025 Dec 14;14(12):1501. doi: 10.3390/antiox14121501 (PMC12729385; doi:10.3390/antiox14121501)
Supplement: Supplementary file 1 [file antioxidants-14-01501-s001.zip › antioxidants-3963323-supplementary.pdf]

Supplementary materials

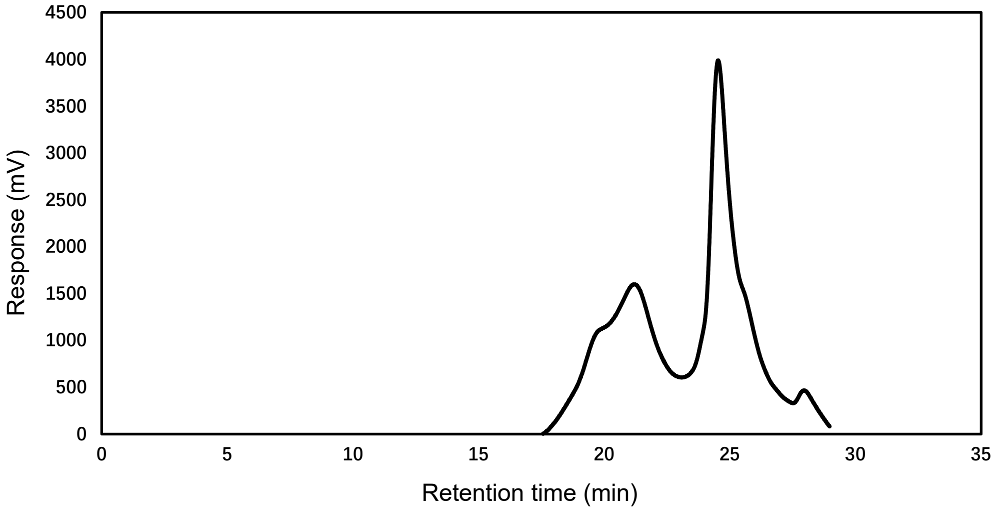

Figure S1. High-performance gel permeation chromatography (HPGPC) profile of EPS-LM.

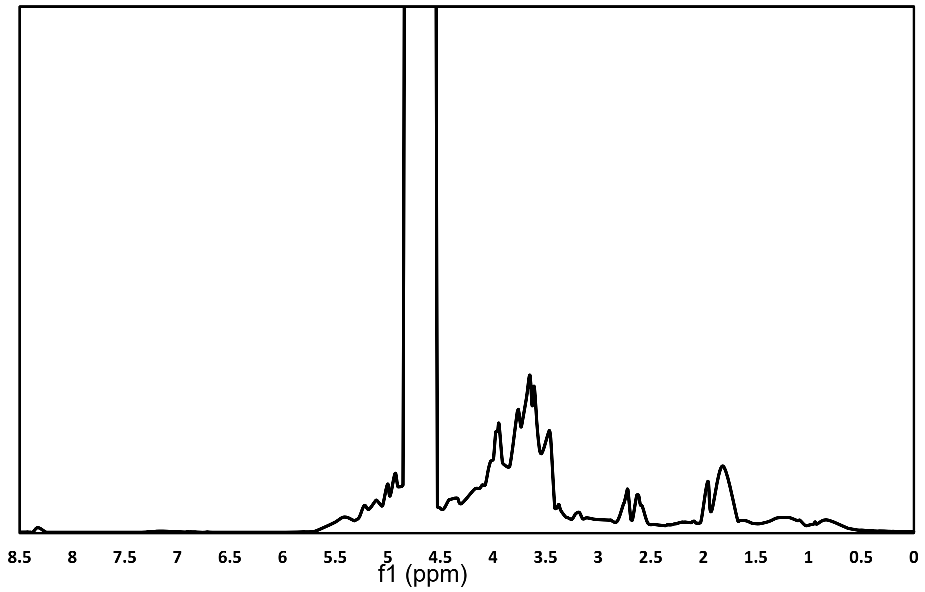

Figure S2. ^{1}H NMR spectrum of EPS-LM.

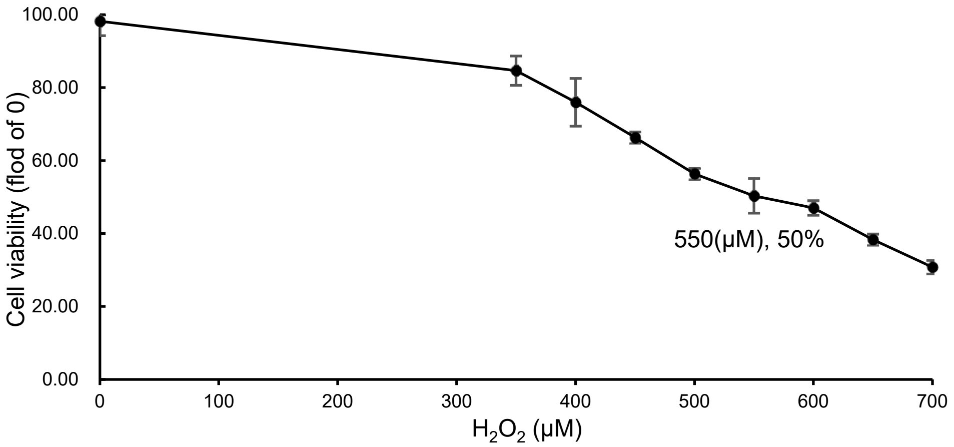

Figure S3. Preliminary dose-response analysis of H_2O_2-induced reduction in Caco-2 cell viability within the concentration range of 0–700 μM. Data are presented as mean ± SEM (n = 6).

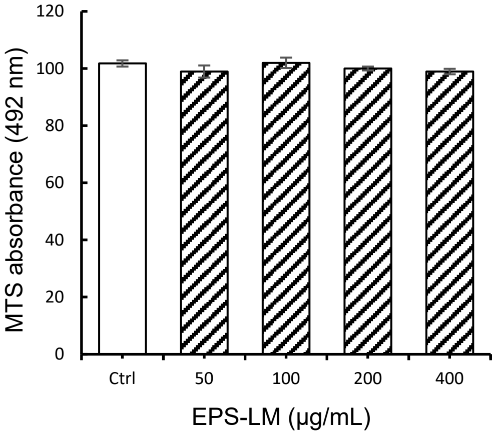

Figure S4. Assessment of EPS-LM interference with the MTS assay in cell-free conditions. Cell-free wells containing culture medium, MTS/PMS and EPS-LM (50-400 μg/mL) were processed under identical conditions. Data are presented as mean ± SEM (n = 6). ns, not significant vs. Ctrl.

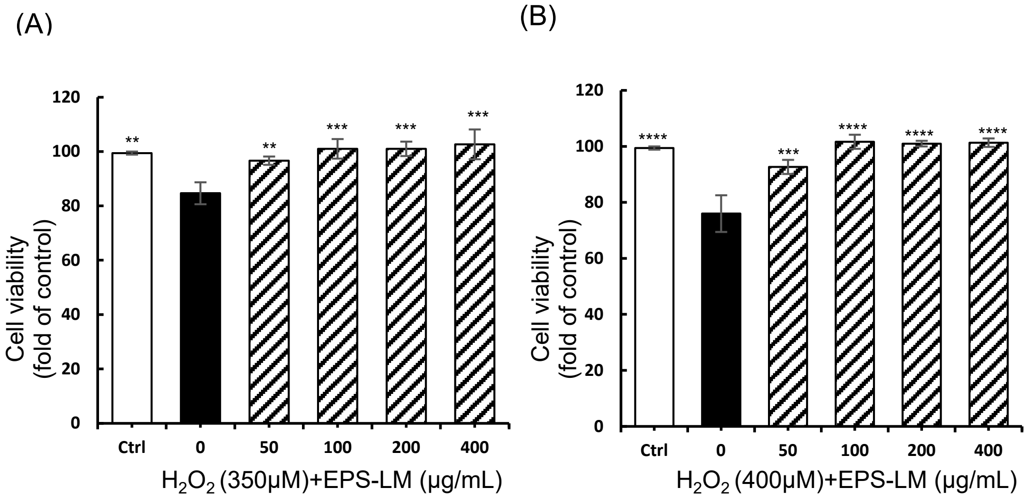

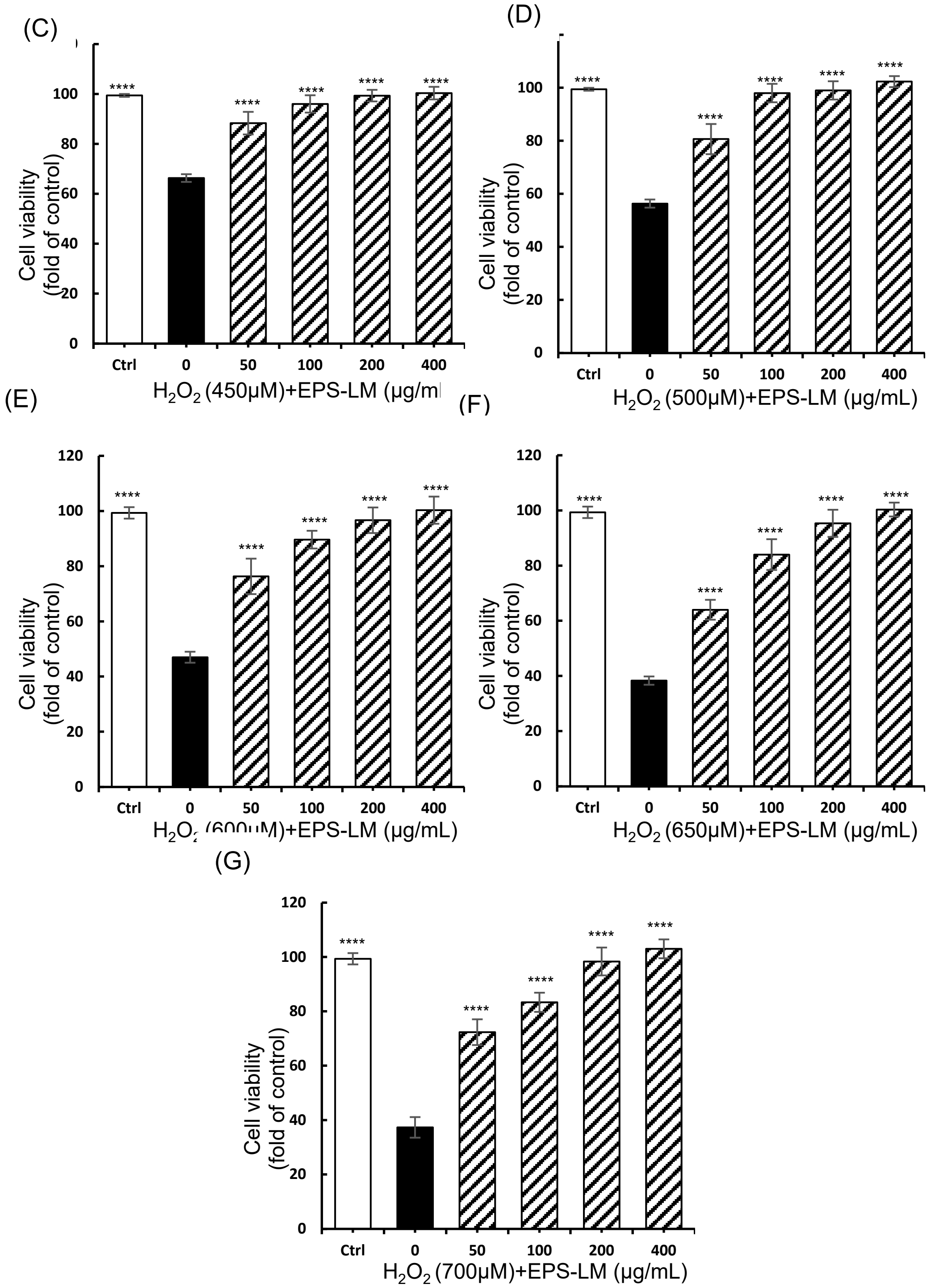

Figure S5. Protective effect of EPS-LM on Caco-2 cell viability under H_2O_2-induced oxidative stress at concentrations ranging from 350 μM to 700 μM (E-G). Data are presented as mean ± SEM (n = 6). (**$p < 0.01$, ***$p < 0.001$, ****$p < 0.0001$ vs. H_2O_2).
